# Efectos del trabajo de pie en trabajadores del sector sanitario

**DOI:** 10.15649/cuidarte.1790

**Published:** 2021-08-20

**Authors:** Jonathan Osorio-Vasco, Yordán Rodríguez

**Affiliations:** 1 Universidad de Antioquia, Facultad Nacional de Salud Pública, Grupo de Investigación Seguridad y Salud en el Trabajo; Medellín; Colombia. Email: jonathan.osorio@udea.edu.co Autor de correspondencia. Universidad de Antioquia Universidad de Antioquia Facultad Nacional de Salud Pública Medellín Colombia jonathan.osorio@udea.edu.co; 2 Universidad de Antioquia, Facultad Nacional de Salud Pública, Grupo de Investigación Seguridad y Salud en el Trabajo; Medellín; Colombia. Email: yordan.rodriguez@udea.edu.co Universidad de Antioquia Universidad de Antioquia Facultad Nacional de Salud Pública Medellín Colombia

**Keywords:** Salud Ocupacional, Posición de Pie, Extremidades Inferiores, Dolor de la Región Lumbar, Dolor Musculoesquelético., Occupational Health, Standing Position, Lower Extremity, Low Back Pain, Musculoskeletal Pain., Saúde do Trabalhador, Posição Ortostática, Extremidade Inferior, Dor Lombar, Dor Musculoesquelética.

## Abstract

**Introducción:**

En el sector sanitario es común la ejecución de actividades de pie; sin embargo, es un tema poco atendido, a pesar de los efectos negativos que se pueden generar en la salud de los trabajadores.

**Objetivo**: Analizar la variación de dolor musculoesqueléticos en las extremidades inferiores y espalda baja, y la variación de volumen en las piernas presentados en trabajadores sanitarios que realizan sus actividades de pie durante una jornada laboral.

**Materiales y métodos:**

Estudio transversal donde se registró la percepción de dolor musculoesquelético de los segmentos corporales: espalda baja y el lado derecho e izquierdo de: muslo-cadera, rodilla, pierna y tobillo- pie y se midió la circunferencia para calcular su volumen de las piernas con la cinta métrica Gulick II a 21 trabajadores del sector sanitario. Los registros y mediciones se realizaron durante la primera y la última hora de una jornada laboral de 8 horas.

**Resultados:**

En todos los segmentos corporales la percepción de dolor musculoesquelético y el volumen en ambas piernas aumentó al final de la jornada laboral respecto al inicio. Los segmentos corporales muslo-cadera izquierda, tobillo-pie derecho y el volumen en ambas piernas aumentaron significativamente.

**Discusión:**

Los resultados obtenidos son similares a estudios con diseños experimentales, con la diferencia que nuestro estudio se desarrolló en condiciones reales sin control de variables.

**Conclusiones:**

Este estudio muestra cómo actividades del sector sanitario ejecutadas durante tiempos prolongados de pie pueden ocasionar en los trabajadores dolor musculoesqueléticos en las extremidades inferiores y la espalda baja, así como un aumento en el volumen de las piernas.

## Abstract (en)

**Introduction:**

In the health care sector, the execution of standing activities is common; however, it is an issue that receives little attention, despite the negative effects that can be generated in the health of workers.

**Objective:**

To analyze the variation of musculoskeletal pain in the lower extremities and lower back, and the variation of volume in the legs in health care workers who perform their activities while standing during a working day.

**Materials and methods:**

A cross-sectional study in which the perception of musculoskeletal pain was recorded in the following body segments: lower back and right and left side of the thigh-hip, knee, leg and ankle-foot, and the circumference was measured to calculate the volume of the legs with the Gulick II tape measure in 21 health care sector workers. Recordings and measurements were taken during the first and last hour of an 8-hour workday.

**Results:**

In all body segments the perception of musculoskeletal pain and the volume in both legs increased at the end of the working day compared to the beginning. The body segments left thigh-hip, right ankle-foot and the volume in both legs increased significantly.

**Discussion:**

The results obtained are similar to studies with experimental designs, with the difference that our study was developed under real conditions without variables control.

**Conclusions:**

This study shows how activities in the health care sector executed during prolonged standing times can cause musculoskeletal pain in the lower extremities and lower back in workers, as well as an increase in the legs volume.

## Resumo

**Introdução:**

No setor de saúde, a execução de atividades em pé é comum; entretanto, é uma questão que recebe pouca atenção, apesar dos efeitos negativos que podem gerar sobre a saúde dos trabalhadores.

**Objetivo:**

Analisar a variação da dor musculoesquelética nos membros inferiores e lombar, e a variação do volume das pernas nos profissionais de saúde que realizam suas atividades em pé durante uma jornada de trabalho.

**Materiais e métodos:**

Estudo transversal no qual foi registrada a percepção da dor musculoesquelética nos segmentos do corpo: região lombar e lado direito e esquerdo da coxa-quadril, joelho, perna e tornozelo-pé, e a circunferência foi medida para calcular o volume das pernas com a fita métrica Gulick II em 21 trabalhadores do setor de saúde. Os registros e medições foram feitos durante a primeira e última hora de um dia de trabalho de 8 horas.

**Resultados:**

Em todos os segmentos do corpo, a percepção da dor musculoesquelética e o volume em ambas as pernas aumentou no final do dia de trabalho em comparação com o início. Os segmentos do corpo coxa-quadril esquerdo, tornozelo-pé direito e o volume em ambas as pernas aumentaram significativamente.

**Discussão:**

Os resultados obtidos são semelhantes aos estudos com desenhos experimentais, com a diferença de que nosso estudo foi realizado em condições reais sem controle de variáveis.

**Conclusões:**

Este estudo mostra como as atividades no setor de saúde realizadas durante períodos prolongados de permanência em pé podem causar dores musculoesqueléticas nos membros inferiores e a região lombar, bem como um aumento no volume das pernas.

## Introducción

Muchas actividades laborales requieren que los trabajadores permanezcan de pie durante gran parte de su jornada laboral: trabajadores de la industria(1), vendedores en tiendas de comercio, supermercados(2),(3) y el personal sanitario(3),(4). En los Estados Unidos se reportó que el 83% de la fuerza laboral industrial permanece en una postura erguida sobre sus dos extremidades inferiores presentando molestias y dolor en los pies(5), en una muestra de población trabajadora australiana se reportó que el 62% trabaja de pie(6), estudios realizados en Quebec (Canadá), muestran que 55,3% de la población trabajadora activa permanece de pie mientras trabaja(7) y se estimó que en el Reino Unido 11 millones de trabajadores permanecen de pie mientras trabajan(8).

La realización de actividades prolongadas de pie durante una jornada laboral puede generar en los trabajadores la aparición de síntomas musculoesqueléticos relacionados con malestar, dolor y/o fatiga en las extremidades inferiores (pies, piernas, muslos y caderas) y la espalda baja, así como el aumento de la circunferencia y el volumen de las piernas en el segmento de los músculos soleo y gastrocnemio(1),(6),(9),(10). El aumento del volumen de las piernas ocurre por la acumulación del flujo sanguíneo en las piernas y estará influenciado por diversos factores como: la adopción de posturas estáticas o dinámicas(11), el tipo de superficie(12), el tipo de calzado(13) y el tiempo de pie(7),(14),(15).

Las enfermedades musculoesqueléticas afectan globalmente alrededor de 1710 millones de personas y están precedidas por el dolor(16) siendo este un síntoma recurrente dentro de las enfermedades musculoesqueléticas(17). La Asociación Internacional para el Estudio del Dolor (International Association for the Study of Pain, IASP, por sus siglas en inglés), define el dolor como: “Una experiencia sensorial y emocional desagradable asociada a un daño tisular real o potencial, o similar a éste.”(18). Debido a la relación entre el dolor y las enfermedades musculoesqueléticas, en este estudio se analiza el dolor musculoesquelético reportado por trabajadores del sector sanitario en las extremidades inferiores y la espalda baja, y su relación con la variación de volumen en las piernas.

El reporte de dolor musculoesquelético en extremidades inferiores y espalda baja ha estado relacionado con el tiempo prolongado de pie, principalmente de manera estática, durante una jornada laboral(6),(15). El dolor en las extremidades inferiores y la espalda baja puede aumentar o disminuir si se encuentra en una superficie dura o blanda(12), con un calzado plano o inestable(13)(19),(20), en postura estática o en movimiento(11),(21).

Para el registro de signos y síntomas en las extremidades inferiores y espalda baja se han usado variados métodos y técnicas: para los síntomas de dolor percibido, las escalas verbales o auto reporte(22), para el registro de la variación de volumen en las piernas las cintas métricas(12),(13) y a través de la volumetría(23), para medir el flujo sanguíneo la flujometría láser y evaluar la actividad muscular los equipos de electromiografía(1),(7).

En el sector sanitario varias actividades requieren que se realicen de pie. Por ejemplo, en las instituciones sanitarias usualmente el personal de enfermería debe estar de pie durante un alto porcentaje de la jornada de trabajo(20), generando dolor en la espalda baja y las extremidades inferiores(24). En un estudio realizado en una institución sanitaria de Nigeria que contó con la participación de médicos, enfermeros y personal de aseo, reportaron que la espalda baja fue la región corporal con mayor prevalencia de dolor, estando asociado al tiempo de trabajo de pie(25). En las instituciones sanitarias no solo el personal de enfermería o médico realiza su trabajo de pie, sino también el personal de limpieza, farmacéutico, administrativo, entre otros(15).

En Colombia según una consulta formal realizada al Ministerio de Trabajo en el año 2016, no se cuenta con estudios específicos sobre población trabajadora de pie y sus efectos en las extremidades inferiores y la espalda baja del cuerpo(26) y menos aún en el sector sanitario. Por lo que es necesario se realicen estudios que aporten evidencia sobre la magnitud del problema y soporten la generación de acciones efectivas en la prevención de signos y síntomas de las extremidades inferiores y la espalda baja.

El objetivo de este artículo es analizar la variación de dolor musculoesqueléticos en las extremidades inferiores y espalda baja, y la variación de volumen en las piernas presentados en trabajadores sanitarios que realizan sus actividades de pie durante una jornada laboral.

La presente investigación se deriva del proyecto de investigación titulado:“Análisis de los signos, síntomas y factores de riesgo asociados a las extremidades inferiores y espalda baja durante la jornada laboral en trabajadores de una institución sanitaria de Medellín”.

## Materiales y métodos

Estudio observacional de tipo transversal donde se analizaron las diferencias en la percepción del dolor musculoesquelético en la espalda baja y las extremidades inferiores y la variación del volumen de las piernas entre el inicio y final de una jornada laboral de 8 horas en 21 trabajadores de una institución sanitaria de Medellín Colombia. También se analizó la relación entre la diferencia de dolor musculoesquelético de la espalda baja y las extremidades inferiores con la diferencia de volumen de las piernas. Se registraron los datos siguientes: la percepción de dolor musculoesquelético en la espalda baja y el lado derecho e izquierdo de muslo-cadera, rodilla, pierna y tobillo-pie; la circunferencia de las piernas de los trabajadores en su puesto de trabajo y posteriormente se calculó el volumen. Las variables de este estudio fueron: la diferencia entre el inicio y final de los síntomas de dolor musculoesquelético por cada segmento corporal y la variación del volumen en las piernas.

### Selección de los participantes

Los trabajadores seleccionados laboraban en ocho puestos de trabajo de interés para la institución sanitaria. Por cada puesto de trabajo se seleccionaron entre uno y máximo tres trabajadores. En los casos donde se desempeñaban más de tres trabajadores en el puesto, la selección fue aleatoria. Para la participación en el estudio, los trabajadores debían ejecutar sus actividades en alguno de los puestos seleccionados, manifestar su participación voluntaria a través de la firma de un consentimiento informado y estar presentes el día de la recolección de datos.

### Síntomas musculoesqueléticos

Para evaluar los síntomas musculoesqueléticos se le preguntó a los trabajadores que indicaran el nivel de dolor usando la Escala Visual Análoga (EVA)(27), representada en una línea de 0 a 100 mm. El extremo izquierdo de la línea indicaba “no dolor” y el extremo derecho indicaba “máximo dolor”. Fueron evaluados 21 segmentos corporales usando como referencia el mapa del cuerpo de la norma ISO/TS 20646(28), de los cuales se eligieron para el análisis de este estudio las extremidades inferiores: tobillo-pie derecho, pierna derecha, rodilla derecha, muslo-cadera derecha, tobillo-pie izquierdo, pierna izquierda, rodilla izquierda, muslo-cadera izquierda y la espalda baja.

### Medición de la circunferencia de las piernas

Para medir la circunferencia de las piernas (derecha e izquierda), se utilizó la cinta métrica Gulick II(29). La cinta métrica está diseñada para disminuir el error en la medición con base en un (1N) Newton de tensión que se encuentra en un dispositivo de control al momento de tensionar la cinta a través de un resorte, garantizando que la medición tendrá aproximadamente la misma fuerza, a diferencia de las cintas métricas comunes. La cinta métrica permite estandarizar la medición, garantizando que todas las mediciones estarán aproximadamente con la misma fuerza al jalar la cinta métrica ayudando a la precisión de las mediciones(12),(13),(30).

Un estudio que evaluó la confiabilidad de las cintas métricas que emplean resortes para estandarizar la medición, reportó un coeficiente de confiabilidad de 0.97 para la pantorrilla y 0.98 para el tobillo de sujetos sanos y baja precisión relativa de 6.36% para la pantorrilla y 12.49% para el tobillo(23). De igual manera en un estudio de comparación de edema en las piernas empleando la cinta métrica Gulick y un método automatizado de medición optoelectrónico de Pero-System, el resultado entregó que las mediciones del volumen entre los dos métodos tenía una alta correlación con un coeficiente de 0,98 para las piernas y 0,96 para los brazos(31).

### Recolección de la información

Inicialmente se realizaron reuniones con el equipo de investigación buscando evitar posibles errores en la medición, donde se estandarizó el procedimiento de medición con la cinta métrica Gulick II siguiendo las indicaciones del fabricante y el uso de la EVA. Posteriormente se tuvo encuentros con el encargado de la seguridad y salud en el trabajo de la institución sanitaria donde se llevó a cabo el estudio, indicándoles el objetivo de la investigación y el protocolo de medición. Se establecieron las jornadas permitidas por la institución sanitaria para el acceso a los trabajadores, las cuales fueron durante cualquier día de la semana en una jornada de 8 horas. Antes de la aplicación de los instrumentos los trabajadores firmaron un consentimiento informado manifestando la voluntad de participación en el estudio a través de la firma y huella en un consentimiento informado.

Para el registro de las mediciones de la percepción del síntoma de dolor musculoesquelético y la circunferencia de las piernas, los trabajadores fueron llevados a un sitio privado y apartado de su puesto de trabajo. Durante la primera hora de la jornada laboral a los trabajadores se les tomó el peso con una balanza calibrada y la talla con un estadiómetro. Se continuó con la medición de las piernas solicitando a los participantes sentarse en una silla, luego quitarse los zapatos y subirse la bota del pantalón de la pierna derecha para dejarla extendida sobre otra silla de similar altura a la silla donde se encontraba sentado.

Para mejorar la medición de la circunferencia y facilitar el cálculo del volumen de las piernas, se utilizó una tablilla de madera con 6 segmentos de 4 cm cada uno y un marcador de tinta permanente para señalar en la pierna los puntos de referencia para la segunda medición; la tablilla se ubicó desde el maléolo lateral iniciando desde el tobillo hasta 20 cm a lo largo del eje longitudinal del maléolo donde la pantorrilla tiene la circunferencia máxima(12),(13),(32). El mismo procedimiento se realizó para la pierna izquierda.

El registro de la medición de cada segmento de las piernas se registró usando un formato estandarizado. Posteriormente, se registró la percepción de dolor de cada segmento corporal, para ello los trabajadores indicaban el nivel de dolor marcando con una línea roja sobre la EVA. Para la evaluación de los síntomas musculoesqueléticos se usó un mapa del cuerpo al inicio y otro al final de la jornada laboral; de esta forma se evitó que la percepción de dolor referida al inicio pudiera influenciar la percepción de dolor al final de la jornada laboral.

### Cálculo del porcentaje de variación de dolor musculoesquelético

El porcentaje de variación de dolor musculoesquelético se calculó a través de la siguiente fórmula:

ΔD%= ((D2 - D1)/D1) x100

Las variables de la fórmula son: D1 es el síntoma de dolor musculoesquelético al inicio de la jornada laboral, D2 es el síntoma de dolor musculoesquelético al final de la jornada laboral. El porcentaje se calculó para cada segmento corporal analizado.

### Cálculo del volumen de las piernas y su porcentaje de variación

Para el cálculo del volumen de las piernas, con base en los registros de la circunferencia se utilizó la fórmula del cono truncado(12),(13),(31):

V=∑(X2+Y2+XY)/3π

Donde “V” es el volumen de la pierna, “X” es la circunferencia inferior del segmento y “Y” es la circunferencia superior del segmento a 4 cm de distancia de “X”. La fórmula se usó para calcular el volumen del inicio de la jornada laboral (V1) y el volumen final de la jornada laboral (V2). También se calcularon los porcentajes de variación de volumen a través del siguiente cálculo(13):

ΔV%=((V2-V1)/V1) x 100

### Procesamiento estadístico de los datos

Para el análisis estadístico se utilizó el software estadístico SPSS versión 23 (SPSS, Inc. 2012). Se hizo un análisis estadístico descriptivo para las características sociodemoGráficas. Se calcularon las diferencias promedio entre la medición inicial y el final del dolor musculoesquelético percibido para cada segmento corporal y para la variación de volumen en las piernas. Se calculó el porcentaje promedio de la variación del dolor musculoesquelético percibido para cada segmento corporal y el porcentaje promedio de la variación de volumen en las piernas. Se usó la prueba de rangos con signo de Wilcoxon para hallar diferencias significativas de los síntomas de dolor musculoesquelético para cada segmento corporal y la variación de volumen en las piernas.

Se utilizó la prueba de Spearman para la correlación entre los síntomas de dolor percibido en los segmentos del lado izquierdo con la variación de volumen de la pierna izquierda, entre los síntomas de dolor en los segmentos del lado derecho del cuerpo con la variación de volumen de la pierna derecha y la variación de volumen de las piernas con la espalda baja.

### Aspectos éticos de la investigación

Para esta investigación se tuvieron en cuenta los principios éticos fundamentados en la Resolución 8430 de 1993, del Ministerio de Salud de Colombia por la cual se establecen las normas científicas, técnicas y administrativas para la investigación en salud, donde se seguirán los lineamientos establecidos en el artículo 6 que establecen los criterios para la investigación con seres humanos. A los participantes se les solicitó su voluntariedad de participación a través de la firma y huella de un consentimiento informado. La investigación contó con la aprobación del Comité de Ética para la Investigación de la Facultad Nacional de Salud Pública de la Universidad de Antioquia (Medellín Colombia) a través del registro número CI 00479 - 2018 aprobado durante la sesión 202 del 16 de noviembre de 2018.

## Resultados

En total participaron en el estudio 21 trabajadores (12 mujeres y 7 hombres), con edad promedio de 35,19 años (DS=9.91); talla media de 165.4 cm (DS=9.91); peso promedio 71.82 kg (DS=12.64). La pierna dominante entre los participantes fue la derecha, reportada por 19 trabajadores.

En la Tabla 1 se presenta la diferencia promedio, el porcentaje de aumento de dolor y la diferencia significativa entre el inicio y al final de la jornada laboral de los segmentos corporales evaluados. Se destaca que todos los segmentos tuvieron un aumento respecto a la medición del inicio de la jornada laboral hasta el final. El segmento corporal que tuvo mayor diferencia de variación fue el muslo-cadera derecha seguido del tobillo-pie derecho y la pierna derecha. Los segmentos corporales con mayor porcentaje de variación fueron tobillo-pie derecho, tobillo-pie izquierdo y el muslo-cadera derecha. Los segmentos corporales con diferencias significativas entre el inicio y final de la jornada laboral fueron: muslo-cadera izquierda (Z=-2,273; p=0,023) y tobillo-pie derecho (Z=-2,395; p=0,017).

La variación de volumen entre el inicio y final de la jornada laboral para la pierna derecha y la pierna izquierda se pueden observar en la Gráfica 1 y la Tabla 2. En la Gráfica 1 se puede notar que ambas piernas aumentaron su volumen desde el inicio hasta el final de la jornada laboral siendo la pierna derecha la de mayor volumen al inicio de la jornada laboral y la que mayor volumen presentó al final de la jornada laboral. En la Tabla 2 se destaca que la pierna derecha tuvo más aumento de volumen promedio en el final de la jornada laboral y el mayor porcentaje promedio de aumento entre el inicio y el final de la jornada laboral fue el de la pierna izquierda con un 2,7%; 0,1% más que la pierna derecha. El aumento del volumen en la pierna derecha (Z=-3,1446; p=0,002) e izquierda (Z=-3,528; p=0,001) entre el inicio y final de la jornada laboral tuvieron diferencias significativas.

**Tabla 1:** Variación promedio de dolor musculoesquelético y porcentaje de aumento del dolor entre el inicio y final de la jornada.

Segmento corporal	Lado del cuerpo	**Dolor *X* inicial**	**Dolor *X final***	**Δ*X***	**ΔD *X* %**	Prueba Wilcoxon	
						Z	P
Muslo cadera	Derecha	11,8	23,85	12,05	102,12	-1,887	0,059
	Izquierda	11,85	18,04	6,19	52,24	-2,273	0,023
Rodilla	Derecha	18,38	20,19	1,81	9,85	-0,489	0,625
	Izquierda	12,85	13,23	0,38	2,96	-0,551	0,582
Pierna derecha	Derecha	13,47	22,04	8,57	63,62	-1,373	0,170
	Izquierda	12,66	13,85	1,19	9,4	-0,523	0,533
Tobillo pie	Derecho	3,61	14,85	11,24	311,36	-2,395	0,017
	Izquierdo	2,7	9,5	6,8	251,85	-1,122	0,262
Espalda baja	-	21,9	26,47	4,57	20,87	-0,513	0,608

Fuente: elaboración propia a partir de los registros de la evaluación del dolor. P<0.05, (n=21).

**Gráfica 1:**
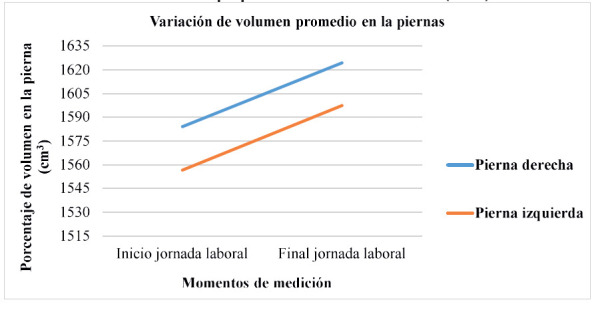
Variación de volumen promedio en las piernas entre el inicio y final de la jornada laboral.

Fuente: elaboración propia con base en los resultados (n=21).

**Tabla 2:** Variación promedio de volumen en las piernas, diferencia promedio, porcentaje promedio de variación y prueba de Wilcoxon.

Pierna	**Volumen inicial *X* cm** ^3^	DS	**Volumen final *X* cm3**	DS	**Δ*X* cm3**	**ΔV *X* %**	Prueba Wilcoxon		
							Z	P
Derecha	1584,2271	245,74	1624,4705	250,87	40,24	2,60	-3,1446	0,002
Izquierda	1556,6319	243,58	1597,5876	245,68	40.95	2,70	-3,528	0,001

Fuente: elaboración propia con base en los registros de la medición del volumen de las piernas. P<0.05, (n=21).

No hubo correlaciones significativas entre la diferencia de dolor percibido de los segmentos corporales del lado derecho del cuerpo y la variación de volumen en la pierna derecha. El mismo resultado se obtuvo con los segmentos corporales del lado izquierdo del cuerpo y la variación del volumen en la pierna izquierda. Los resultados pueden observarse en la Tabla 3.

**Tabla 3:** Correlación de Spearman entre la diferencia de dolor de los segmentos corporales y la diferencia de volumen de las piernas por cada lado del cuerpo.

Segmento corporal	Lado del cuerpo	Volumen pierna derecha			Volumen pierna izquierda	
		Rho	P	Rho	P
Muslo cadera	Derecha	0,332	0,142	-	-
	Izquierda	-	-	0,368	0,101
Rodilla	Derecha	-0,292	0,199	-	-
	Izquierda	-	-	0,161	0,485
Pierna derecha	Derecha	0,080	0,731	-	-
	Izquierda	-	-	0,173	0,454
Tobillo pie	Derecho	0,167	0,470	-	-
	Izquierdo	-	-	0,081	0,727
Espalda baja	-	-0,179	0,438	0,062	0,790

Fuente: elaboración propia a partir de los registros de la medición del dolor y de volumen de las piernas. P<0,05, R>0,25, (n=21).

## Discusión

En este estudio se evidenció un aumento de dolor musculoesquelético de las extremidades inferiores y la espalda baja entre el inicio y final de la jornada laboral (Tabla 1). Los segmentos corporales con mayor porcentaje de variación fueron el tobillo-pie derecho, el tobillo-pie izquierdo y el muslo-cadera derecha los cuales aumentaron por encima del 100% (Tabla 1). Los segmentos corporales tobillo-pie derecho (Z=-2,395 p=0,017) y el muslo-cadera derecha (Z=-2,273; p=0,023) presentaron diferencias significativas entre la primera y última medición (Tabla 1). Diferentes investigaciones analizadas en el estudio de revisión científica hecha por Peter Coenen y otros, compilaron información que muestra cómo el reporte de dolor en los tobillos, los pies, piernas y en general las extremidades inferiores crece entre las dos primeras horas(6). Este estudio analizó la variación de la percepción de dolor que se produjo durante aproximadamente 8 horas de una jornada de trabajo cotidiana, donde no se manejaron intervenciones como tapetes antifatiga y calzado cómodo o inestable.

El volumen de las piernas derecha (Z=-3,1446; p=0,002) e izquierda (Z=-3,528; p=0,001) tuvieron diferencias significativas entre la primera y última medición (Tabla 2). Se puede observar (figura 1) como la variación del volumen de ambas piernas aumenta. Llama la atención que a pesar de que la pierna derecha fue elegida mayoritariamente como la dominante por los participantes (19 de 21 participantes en el estudio) y la que más volumen presenta entre el inicio y final de la medición, no tiene el mayor porcentaje de crecimiento, pues la pierna izquierda creció un 0,10% promedio más que la pierna derecha (Tabla 2). El hallazgo de la variación de volumen en las piernas fue también evidenciado en un estudio que midió la variación en diferentes tipos de superficie mostrando aumento en cada una de ellas durante una jornada laboral de 8 horas(12), otra investigación que estudió la variación de volumen en diferentes tipos de calzado mostró aumento del volumen de las piernas con cada uno de los tipos de calzado estudiados(13), otro estudio valoró el volumen de las piernas en 3 diferentes posturas: estar de pie, sentado, de pie y sentado durante periodos de tiempo de 90 minutos. En las tres condiciones el volumen de las piernas aumentó(3).

El resultado presentado en este estudio sobre la variación del volumen de ambas piernas no se distancia de lo reportado en investigaciones anteriores, la diferencia se encuentra en que la toma de registro del volumen incluyó dos segmentos más de las piernas (20 cm desde el tobillo hasta la parte superior de la perna) que lo presentado en la investigación de Zander, King, and Ezenwa (2004) que solo incluyó 3 segmentos (12 cm desde el tobillo hasta la parte superior de la pierna)(12) y respecto a los otros dos estudios, la diferencia se encuentra en que la medición del volumen se llevó a cabo durante el desarrollo normal de la jornada laboral donde cada trabajador se encontraba en su puesto de trabajo mientras que estos estudios fueron diseñados con condiciones controladas(3),(13). El resultado del aumento de volumen puede estar influenciado por diferentes factores de riesgos ocupacionales (como el tiempo en permanecer de pie, tiempos de descanso y recuperación, tipo de calzado, uso de maquinaria con las piernas, posturas corporales y tipos de superficie) y/o individuales (como la edad, el peso, la talla e índice de masa corporal). En el caso particular de este estudio, los trabajadores siempre estuvieron sobre una superficie dura (baldosa). Estudios posteriores deberán permitir identificar qué factores de riesgo influyen de manera simultánea para que haya variación en el volumen de las piernas.

En este estudio no se hallaron correlaciones entre el aumento del volumen de las piernas (entre el maléolo y la pantorrilla) y el dolor en los segmentos corporales de las extremidades inferiores y la espalda baja, a pesar de que el síntoma de dolor aumentó en todos los segmentos corporales analizados y el aumento del volumen de ambas piernas. Lo hallado por Antle y otros en 2013, demostró que el aumento del volumen en el pie y el soleo tuvo fuerte correlación (R>0,8) con molestias en los pies y las rodillas(14). Otros estudios sugieren que la aparición de síntomas de dolor, malestar o fatiga en las extremidades inferiores pueden estar relacionados con condiciones vasculares como el aumento del volumen, mientras que en la espalda baja la aparición de síntomas puede ser multifactorial donde variables vasculares, musculares y posturales pueden estar presentes(4),(7).

### Limitaciones

Una de las limitaciones de este estudio fue el reducido tamaño de muestra (21 participantes). Esta muestra no es representativa de la población laboral del sector sanitario que trabaja de pie, por lo que los resultados reportados deben ser valorados con precaución. Sin embargo, este estudio al ser realizado en un contexto real de trabajo es valioso, ya que la mayoría de los estudios consultados sobre los efectos del trabajo de pie se han realizado en condiciones experimentales(3),(7),(12),(14),(33),(34) y con tamaños de muestra incluso menores al estudio que se presenta en este artículo, por ejemplo: 18 participantes(3), 10 participantes(7), 13 participantes(12), 18 participantes(14), 15 participantes(33) y 16 participantes(34).

Por otro lado, las mediciones en los estudios experimentales se han realizado durante periodos cortos de tiempo (90 minutos(3), 32 minutos(7), 34 minutos(14), 34 minutos(33), 34 minutos(34)), lo cual limita la extrapolación de los resultados de los efectos de permanecer de pie a contextos reales de trabajo (jornada laboral de ocho o más horas de trabajo)(7),(33),(34). A raíz de esto, algunos autores han planteado la necesidad de realizar estudios en contextos reales que permitan contrastar los resultados obtenidos con los reportados en condiciones experimentales(7),(33),(34). En este sentido, una fortaleza a destacar en nuestro estudio es que aporta evidencia sobre los efectos del trabajo de pie en una jornada de trabajo de ocho horas.

Otra limitación de nuestro estudio, es que se centró en el análisis de la variación de dolor musculoesquelético y la variación de volumen en las piernas durante la jornada laboral, y no en estudiar las causas o factores que provocan estos efectos. Por lo que recomendamos que en futuros estudios en contextos reales se incluyan en el análisis los posibles factores de riesgo y variables mediadoras, pues al estar en un contexto real es difícil controlar todas las variables(12).

También se recomienda aumentar el tamaño de la muestra, donde se incluya mayor variedad de actividades laborales que se realizan de pie. No obstante, debe considerarse que la realización de estudios en contextos reales, como el presentado en este artículo, estará limitado por la voluntariedad y disponibilidad de los participantes debido a las restricciones de tiempo, carga de trabajo, complejidad del servicio sanitario, así como la aprobación por parte de los directivos de las instituciones de salud e interés en la temática.

## Conclusiones

Este estudio muestra como actividades del sector sanitario que se ejecutan durante tiempos prolongados de pie pueden ocasionar síntomas musculoesqueléticos de dolor en las extremidades inferiores y la espalda baja, así como aumento de volumen de las piernas en los trabajadores. Los segmentos corporales con diferencias significativas con presencia de síntoma de dolor musculoesquelético fueron: muslo-cadera izquierda y tobillo-pie derecho. La variación del volumen de ambas piernas tuvo cambios significativos. A pesar de que los síntomas de dolor musculoesquelético en todos los segmentos corporales aumentaron, no se encontró correlación con la variación del volumen.

Los resultados obtenidos sugieren que se deben incrementar las acciones preventivas con el fin de reducir los efectos negativos en la salud que ocasiona el trabajo de pie, así como la necesidad de incrementar esfuerzos investigativos en esta temática.
